# Community Level Offset of Rain Use- and Transpiration Efficiency for a Heavily Grazed Ecosystem in Inner Mongolia Grassland

**DOI:** 10.1371/journal.pone.0074841

**Published:** 2013-09-18

**Authors:** Ying Z. Gao, Marcus Giese, Qiang Gao, Holger Brueck, Lian X. Sheng, Hai J. Yang

**Affiliations:** 1 Key Laboratory of Vegetation Ecology, Northeast Normal University, Changchun, Jilin, China; 2 Institute for Crop Production and Agroecology in the Tropics and Subtropics, University of Hohenheim, Stuttgart, Baden-Württemberg, Germany; 3 College of Resources and Environment, Jilin Agricultural University, Changchun, Jilin, China; 4 State Environmental Protection Key Laboratory of Wetland Ecology and Vegetation Restoration, Northeast Normal University, Changchun, Jilin, China; United States Department of Agriculture, United States of America

## Abstract

Water use efficiency (WUE) is a key indicator to assess ecosystem adaptation to water stress. Rain use efficiency (RUE) is usually used as a proxy for WUE due to lack of transpiration data. Furthermore, RUE based on aboveground primary productivity (RUE_ANPP_) is used to evaluate whole plant water use because root production data is often missing as well. However, it is controversial as to whether RUE is a reliable parameter to elucidate transpiration efficiency (TE), and whether RUE_ANPP_ is a suitable proxy for RUE of the whole plant basis. The experiment was conducted at three differently managed sites in the Inner Mongolia steppe: a site fenced since 1979 (UG79), a winter grazing site (WG) and a heavily grazed site (HG). Site HG had consistent lowest RUE_ANPP_ and RUE based on total net primary productivity (RUE_NPP_). RUE_ANPP_ is a relatively good proxy at sites UG79 and WG, but less reliable for site HG. Similarly, RUE_ANPP_ is good predictor of transpiration efficiency based on aboveground net primary productivity (TE_ANPP_) at sites UG79 and WG but not for site HG. However, if total net primary productivity is considered, RUE_NPP_ is good predictor of transpiration efficiency based on total net primary productivity (TE_NPP_) for all sites. Although our measurements indicate decreased plant transpiration and consequentially decreasing RUE under heavy grazing, productivity was relatively compensated for with a higher TE. This offset between RUE and TE was even enhanced under water limited conditions and more evident when belowground net primary productivity (BNNP) was included. These findings suggest that BNPP should be considered when studies fucus on WUE of more intensively used grasslands. The consideration of the whole plant perspective and “real” WUE would partially revise our picture of system performance and therefore might affect the discussion on the C-sequestration and resilience potential of ecosystems.

## Introduction

Water use efficiency (WUE) of plants is defined as being the net carbon gain per unit of water lost and considered as one key-parameter for measuring ecological functionality in semiarid ecosystems [1]–[2]. The net primary productivity of semiarid and arid grasslands in Inner Mongolia steppe ecosystems is primarily limited by water availability [3]–[5]. In this context, the relationship between biomass productivity and water use is crucial in terms of predicting systems’ responses to climate variability and land-use impacts [1], [6]. Water use efficiency can be assessed on different scales, ranging from system approaches to instantaneous values of transpiration efficiency. Rain use efficiency (RUE), the amount of biomass produced per unit precipitation, as a unifying concept is an integral measure for assessing the response of primary productivity to spatio-temporal changes in precipitation and has been widely used for the evaluation of water use in different ecosystems [7]–[9].

Besides natural climate variability, land use changes have potentially the strongest impact on ecosystem functions by altering water, carbon and nitrogen dynamics [10]–[12]. Grazing is the most common land use type in the Inner Mongolia steppe ecosystems which co-developed under natural grazing from an evolutionary perspective and are therefore considered to be adapted to herbivory. However, in the past four decades, the increasing herd sizes have resulted in substantial degradation of the Inner Mongolian grasslands [13] which in turn may have changed ecosystem functions in relation to water use efficiency. Continuous heavy grazing not only alters plant and litter biomass, species composition and canopy structure [4], [14]–[15], but also significantly changes soil physical characteristics, land cover surface and system’s energy balance [11], [16]. Livestock feed intake and trampling decreases canopy rainfall interception and increases the risk of wind erosion, topsoil runoff and high soil evaporation [11], [17]–[18]. These effects modify water pathways by increasing unproductive (i.e., not transpired) water losses, which consequentially reduce RUE at heavily grazed and degraded sites [15].

However, RUE of a particular ecosystem or grassland site does not necessarily reflect the real plant water uptake per unit of biomass produced. The transpiration efficiency (TE), defined as the amount of biomass produced per unit of water transpired by leaves, is therefore used to quantify the plant physiological performance in relation to water use. Different land use types are known to alter plant species composition, leaf and root traits, and root distribution in plant communities, potentially affecting the related transpiration efficiency [2]. To our knowledge there is no systematic study focusing on how land use practices affect TE in semi arid grasslands since the relative partitioning of evapotranspiration (ET) into soil evaporation (E) and plant transpiration (T) involves methodological challenges in separating these two parameters within a plotted water balance.

Most of the assessments of RUE and TE are based on aboveground net primary productivity (ANPP) since firstly ANPP is more easily measured than total net primary productivity (NPP) including the root system and secondly it relates directly to livestock carrying capacity and reduction in erosion risk [15]. However, estimates of RUE and TE based on ANPP may be biased if used as a proxy to estimate total plant biomass productivity and carbon uptake efficiency in relation to water used. Root systems, especially in drought prone areas, are known as considerable carbon sinks, not necessarily corresponding to the carbon uptake of aboveground plant organs [10]. It has been shown that grazing decreased both ANPP and BNPP in semi-arid environments, especially in dry years when resources were limited [5], [15], [19]–[21]. However, the biomass partitioning between shoot and root systems is also affected by grazing with relatively more carbon allocated belowground [15], [22]. For the typical Inner Mongolia steppe, a highly variable carbon allocation to belowground organs has been shown among grassland sites in response to annual rainfall variability and land use modes [10]. In this context, it will be a promising approach to analyze RUE and TE from the whole plant perspective, since this may revise our picture of plant water use efficiency under different land use practices and contrasting climatic (rainfall) conditions. Furthermore, considering the total plant production by including belowground productivity into water use efficiency analyses might help us to reassess grassland ecosystem functions related to fodder productivity and carbon sequestration under future stress scenarios.

The main objectives of this study dealing with plant community RUE and TE at three different managed Inner Mongolia grassland sites within three years of contrasting rainfall amounts were therefore to analyze: i) if RUE based on aboveground net primary productivity (RUE_ANPP_) can be used as a proxy for RUE based on whole plant net primary productivity (RUE_NPP_), ii) if estimates of TE change our view of plant community water use in relation to RUE, and iii) how plant community water use efficiency based on RUE and TE is affected by different land use types and annual rainfall variability.

## Methods

### Ethics Statement

We declare that all necessary permits were obtained for the described field studies. The lands which we used belong to the local government. No specific permissions were required for these lands. The local government encourages the scientific researches like what we did. The study area did not involve endangered or protected species. Dr. Qingmin Pan who is the leader of the Inner Mongolia Grassland Ecosystem Research Station as a representative to issue the permissions to use these lands including a site fenced since 1979 (UG79), a winter grazing site (WG) and a heavily grazed site (HG). We were informed to protect the lands and wildlife during the study.

### Study Area

**Figure 1:**
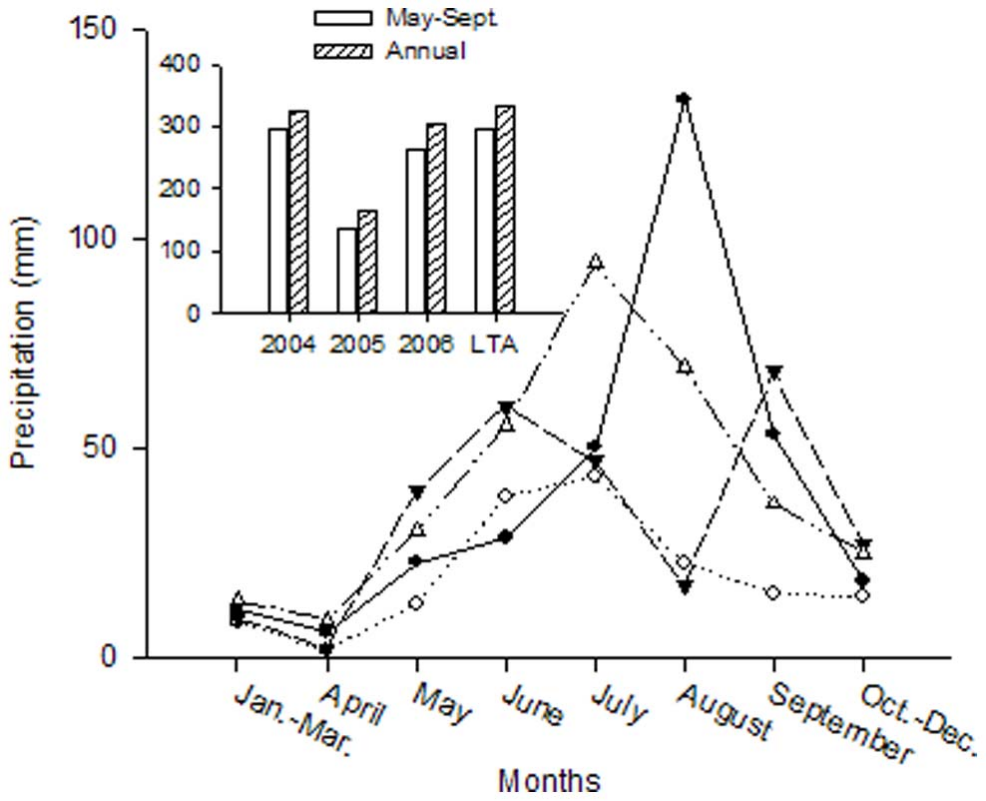
Precipitation (mm) of the experimental site. A solid line (seasonal precipitation in 2004), a dotted line (seasonal precipitation in 2005), a long dashed line (seasonal precipitation in 2006) and a dash-dot-dot line (seasonal long-term average precipitation from 1982 to 2006). The inserted figure showed annual precipitation in 2004, 2005, 2006 and annual long-term average precipitation (LTA) from 1982 to 2006.

The study area was located in the Xilin River watershed (3.800 km^2^) in the Inner Mongolia Autonomous region (43° 32′ N, 116° 40′ E, 1200 m a. s. l.). Based on the last 25-year meteorological data recorded by the Inner Mongolia Grassland Ecosystem Research Station (IMGERS), the growing season lasts about five months from early May to late September. In this period, the monthly average temperature is above 5°C, reaching a maximum of 19°C in July. The winter is dry and extremely cold with a mean temperature in January of −22°C. Main vegetation types are *Leymus chinensis* and *Stipa grandis* communities with a green ground cover of 30–70% at peak biomass time in August and a vegetation height of 0.3 m [4]. Mean annual precipitation (MAP) was 348 mm for the period 1979–2002 with more than 85% of precipitation occurring between May and September reaching a maximum in July (Figure 1). The three experimental years contrasted each other in terms of rainfall and seasonal distribution. In 2004 the amount of rainfall was typical (325 mm) and 2005 was the driest year ever recorded at IMGERS with only 142 mm of rainfall between May and September with an annual total of 166 mm. 2006 was a year with nearly normal rainfall amount (304 mm) but no pronounced summer rainfall peak and considerable rainfall as a snow event (approximate 50 mm) at the end of the growing season in September. Although the most rainfall occurs during the growing period, plant water uptake is normally limited by both seasonal and/or annual rainfall fluctations and the strong evaporative rate. Evaporation was recorded using a standard class A evaporation pan and reached annual values of up to 1750 mm yr^−1^, resulting in water deficits per year as large as 1400 mm.

The experiment was conducted at three different sites characterized by different land-use types. The first site of 24 hectares has been fenced since 1979 (designated as UG79) in order to set up a bench mark area of maximum conservation. Land use before that time was grazing at a low to moderate intensity. Dominant plant species from 2004 to 2006 were the perennial rhizome grasses *Leymus chinensis* and *Carex korshiskyi*, the perennial bunch grasses *Stipa grandis* and *Achnatherum sibiricum* and the shrub *Caragana microphylla* (Table 1). The second site of 40 ha (approximately 600 m apart from site UG79) representing a moderate land use intensity was used for haymaking and winter grazing since 2000 (designated as WG). Before 2000, the area was continuously grazed at a low to moderate intensity (1 sheep unit per hectare). *Leymus chinensis* and *Stipa grandis* were dominant during the experimental years from 2004 to 2006 and almost no herbaceous species were present. The third site of 100 ha has been heavily grazed for the past 30 years (designated as HG). The site is located about 2.5 km northeast of the other two sites. The average grazing intensity over the last 10 years has been more than 2 sheep units per hectare. Grazing intensity expressed in terms of feed intake was about 50–60% in 2004 and 70–80% of aboveground net primary productivity in 2005 and 2006. This site was co-dominated by the perennial rhizome grasses *Carex duriuscula* and *Agropyron michnoi*, the perennial bunchgrasses *Stipa grandis* and *Cleistogenes squarrosa*, the perennial forb *Potentilla acaulis* and the semishrub *Artemisia frigida* (Table 1). Annual herbaceous species such as *Chenopodium* spp. appear frequently depending on rainfall amount and distribution.

**Table 1:** Species composition, topsoil characteristics (0–4 cm depth, except texture 0–10 cm depth) and average growing season soil water content of Inner Mongolia typical steppe under different land use modes.

Parameters	UG79	WG	HG
**Species composition**	*Leymus chinensis, Carex korshinskyi*,*Stipa grandis Achnatherum sibiricum*,*Caragana microphylla*	*Leymus chinensis*, *Stipa grandis,* *Cleistogenes squarrosa,* *Carex korshiskyi*	*Artemisia frigida, Potentilla acaulis, Cleistogenes squarrosa, Carex duriuscula*
**Topsoil characteristics**			
Organic carbon (mg g^−1^)	31.0±5.5	25.9±4.5	17.0±4.2
Total N (mg g^−1^)	3.15±0.50	2.72±0.44	1.74±0.39
C:N ratio	9.8±0.3	9.5±0.4	9.7±0.4
Soil texture (mg g^−1^)			
Clay	220±10	270±10	180±10
Silt	180±40	280±40	180±20
Sand	600±41	500±40	640±30
Bulk density (g cm^−3^)	0.94±0.10	1.09±0.08	1.28±0.08
pH (CaCl_2_)	6.6±0.24	6.7±0.29	6.6±0.25
**Soil water content (V%)**			
2004	18.8	17.6	13.5
2005	10.2	9.0	6.4
2006	12.6	11.6	10.4

*Notes:* Mean values (± SD) from grid samplings in 2004; HG and UG79 n = 98 samples, WG n = 122 samples. Data were compiled from Steffens et al. [18], Giese et al. [12], Gao et al. [4] and Krümmelbein et al. [24]. Average soil water content was measured at 30-minute intervals from May through September at 5 cm depth.

UG79: ungrazed site since 1979; WG: winter grazing site; HG: heavy grazing site.

Soils of the study sites were classified as calcic Chernozems with a sandy loam texture (Table 1). Top soil carbon and nitrogen content decreased with increasing land use intensity and bulk density was lowest in the long term protected site. Soil water content was measured at a 5 cm depth with three replicates on each site during the growing season at 30 minute intervals with calibrated Theta probes (Type ML2x; Delta-T Devices, Cambridge, UK). Throughout the years from 2004 to 2006, site UG79 had higher soil water content compared to the grazed sites; the dry year in 2005 reduced average soil water content at all sites by around 50% with carryover effects into 2006 (Table 1). Groundwater levels in farm wells close to experimental sites were below 8 m from the soil surface, indicating that capillary rise or leaching was most likely not contributing to site water balance.

Soil types and species composition of the experimental sites were similar before fencing (personal communation with famers who lived there since 1950) [23]. Detailed information from the three sites of species composition, soil physical parameters and nutrient availability were described by Krümmelbein et al. [24], Steffens et al. [18], Gao et al. [4], and Giese et al. [25].

### Measurements

#### Vegetation sampling

Five representative plots of 30×40 m were established on each site for measurements of above- and belowground biomass and leaf area index. These plots were systematically selected, taking topography and spatial coverage of the given land use type into consideration. As recommended by Zhou et al. [26], it is necessary to consider big size plots due to the potential spatial heterogeneity of vegetation pattern and resources. Within each plot, plant material was cut down to soil surface from 1×1 m squares with three replications at three sites during the three years. Before clipping, litter on the soil surface was collected with a hand rake. The biomass was first separated into green plant parts and dead material. Shrubs and semi-shrubs (for example *Caragana microphylla* and *Artemisia frigida*) were clip-harvested in mid-August and biomass of new grown branches were considered to represent ANPP. All samples were dried at 75°C to a constant weight.

Aboveground net primary productivity (ANPP) of sites UG79 and WG, which were not grazed during the vegetation period, was estimated by peak aboveground green biomass (mid-August). For site HG, ANPP was estimated from the final residual live biomass plus the estimates of consumption by sheep during the growing season. Sheep consumption was estimated as the difference between biomass measured inside and outside of sheep exclosure cages of 1.5 m×1.5 m size with ten replicates at each site.

Root samples for each year were taken with a soil auger down to a depth of 1 m after aboveground biomass was removed in each plot. The soil column was separated into soil depth intervals of 0–10 cm, 10–20 cm, 20–50 cm, 50–70 cm and 70–100 cm. Belowground net primary productivity (BNPP) was determined by soil auger sampling in 2004 and the ingrowth core method in 2005 and 2006. A detailed description of root sampling methods is given by Gao et al. [10].

Rain-use efficiency (RUE, g m^−2^ mm^−1^) was calculated from the ratio of ANPP and NPP respectively to annual precipitation. Transpiration efficiency (TE, g m^−2 ^mm^−1^) was defined as the ratio of ANPP and NPP respectively to transpiration during the growing season from May to September.

#### Stable carbon isotope measurements

Leaf and bulk shoot material of the most abundant species *Leymus chinensis*, *Stipa grandis*, *Cleistogenes squarrosa*, *Potentilla acaulis*, *Artemisia frigida* was collected in July 2005 for carbon isotope measurements from the three sites by taking samples from a 0.25 m×0.25 m quadrat in ten replicates. All samples were ground in a ball mill and analyzed with a continuous-flow Delta C mass spectrometer (FinniganMAT, Bremen, Germany) for carbon isotope signature. The internal standard was CO_2_ which was calibrated against a urea reference (isotopic ratio: −49.44%).

#### Estimation of water balance components with HYDRUS-1D

Data of soil water content, bare soil evaporation, and latent and sensible heat balances were monitored and components of the field water balance were estimated with the HYDRUS-1D model [27]. HYDRUS-1D was parameterized and then validated by field observations. The evapotranspiration was estimated from the FAO Penman–Monteith equation [28]. The potential evaporation and transpiration was partitioned according to Beer’s Law as a function of the cover area index of material covering the soil. In this equation, the estimation of potential transpiration was based on detailed field leaf area index measurements. Based on field measured root length density and root distribution date, root water uptake was simulated using the model of Feddes et al. [29]. Combining the soil water transport model with the plant growth model is vital to get a reasonable partitioning of calculated ET into actual evaporation and plant transpiration. Details of parameterization and validation of the model are described by Zhao et al. [11].

### Statistical Analysis

Due to statistical limitations of a pseudoreplicated experimental design, error variance of pseudo-replications is small compared to the error variance estimated on (true) replications. The real problems with pseudoreplication are that 1) confidence intervals are too small and, 2) this increases the probability of a Type I error. Therefore, the experiment’s results based on pseudoreplicated design are preliminary and exploratory, and our any discussions based on these concepts are given in the precautionary tone.

Year (Y) and site (S) effects on RUE_ANPP_, RUE_NPP_, TE_ANPP_ and TE_NPP_ were tested by Y*S 2-factor interaction in analysis of variances (ANOVA) using SPSS version 13.0. Furthermore, the Kruskal-Wallis test was performed with the open source software R to analyze differences among means of RUE_ANPP_, RUE_NPP_, TE_ANPP_ and TE_NPP_ and carbon isotope discrimination (δ^13^C ‰) of dominant species. The coefficient of variation was calculated as: CV = (standard deviation/mean)×100. Linear regression analyses were used to evaluate the relationship of RUE_ANPP_ with RUE_NPP_ and TE_ANPP_, RUE_NPP_ with TE_NPP_, litter with T/ET and TE with Delta ^13^C (‰).

As long-term factorial grazing experiments were not established in this region and the supply of equipment and people power to perform simultaneous measurements on several sites were unrealistic, a pseudoreplicated experimental design was unavoidable. Site selection was done in close cooperation with the Institute of Botany, Chinese Academy of Sciences, providing a long-term experience of experimental fieldwork on these particular sites of which many results were published. Unlike other studies which focus on (short-term) grazing effects on homogenous field plots within factorial experiments, effects of land use presented and discussed here are considered to be representatives for sites under different long-term grazing regimes in the area. However, we can’t exclude the fact that site effects may have influenced our results and therefore the information provided should be carefully considered in terms of upscaling and transferability to other grassland sites and ecosystems.

## Results

### Estimation of Water Balance Components

Water flux via surface runoff, drainage and interception represented only 6–12% of the total seasonal water balance at the 3 sites (Table 2). Interception was much higher at sites UG79 and WG than at site HG, while surface runoff and drainage was much lower at sites UG79 and WG than at site HG. Most of the annual precipitation at the different sites was fluxed by evapotranspiration (ET), in the range of 86–101% of the annual precipitation received. The relative portion of plant transpiration to surface evaporation varied considerably between sites and years. This water pathway was highest at site UG79 throughout all the years varying between 45.5% (2005, 2006) and 63.6% (2004) of the annual precipitation received. Water consumption by plant transpiration was lowest at site HG ranging from 23.5% (2006) to 48.5% (2004), while site WG showed intermediate values ranging from 41.7% (2006) to 55.3% (2004). Relative to transpiration, evaporation increased considerably in the two dry years (2005 and 2006) at site HG, while at sites UG79 and WG the shift to evaporation was less pronounced.

**Table 2:** Simulated water budget components during growing periods in 2004–2006, inverse simulation results with Hydrus-1D.

Year	Treatment	*I*	*T*	*E*	*ΔS*	*D*	*R*	*Error*	*ET*	*T/E*
2004	UG79	5.9	63.6	37.8	−8.9	0.3	0.6	0.6	101.4	1.68
(*P* = 275)	WG	5.3	55.3	42.3	−6.5	1.1	1.5	1.1	97.6	1.31
	HG	2.6	45.5	51.7	−4.8	2.5	2.0	0.5	92.7	0.88
2005	UG79	10.1	48.5	40.4	0.9	1.6	0.1	−1.6	88.9	1.20
(*P* = 147)	WG	9.0	44.0	47.1	1.1	0.8	0.1	−2.1	91.1	0.93
	HG	4.5	27.4	61.6	5.0	3.5	0.3	−2.4	89.0	0.44
2006	UG79	7.6	48.5	41.4	3.0	0.5	0.3	−1.3	89.9	1.17
(*P* = 242)	WG	6.7	41.7	49.8	2.7	0	0.5	−1.5	91.5	0.84
	HG	3.4	23.5	62.7	6.9	0.8	1.9	0.8	86.2	0.37

(in % of annual received precipitation, *P*: Precipitation, *I*: Interception, *T*: Plant transpiration, *E*: Soil evaporation, *ΔS*: Water storage change, *D*: Drainage, and *R*: Runoff; *Error* is the water balance model error; *ET*: Evapotranspiration) and the ratio of *T* to *E* (*T/E*). Symbols for different sites as in Table 1.

### Land use and Drought Effects on RUE and TE

**Figure 2:**
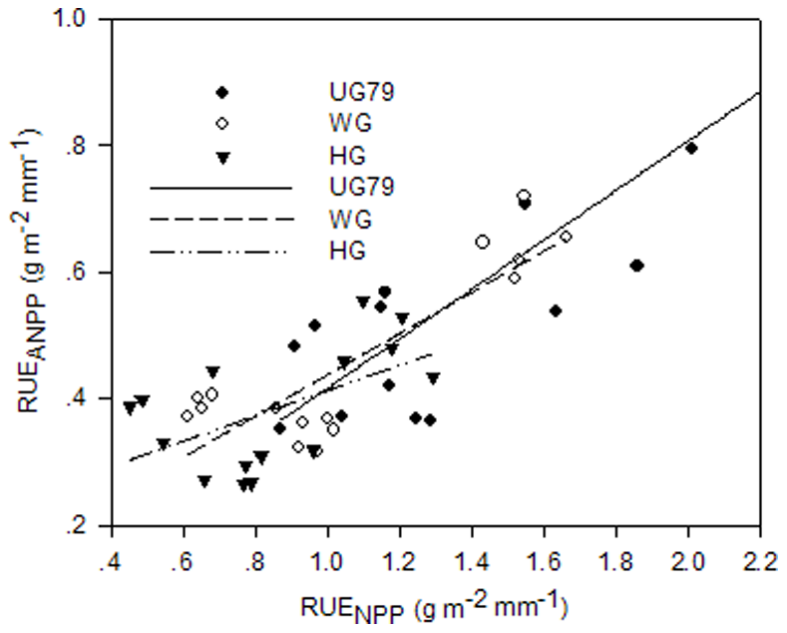
Relationship between RUE_ANPP_ (g m^−2 ^mm^−1^) and RUE_NPP_ (g m^−2 ^mm^−1^). The data were from three differently managed sites in 2004, 2005 and 2006. The regression equations for UG79: y = 0.025+0.391 x, r^2^ = 0.673, n = 15, P<0.001; for WG: y = 0.112+0.326 x, r^2^ = 0.742, n = 15, P<0.001; for HG: y = 0.213+0.201 x, r^2^ = 0.306, n = 15, P = 0.029. UG79 =  ungrazed site since 1979; WG = winter grazing site; HG = heavy grazing site; RUE_ANPP_ = rain-use efficiency based on aboveground net primary productivity; RUE_NPP_ = rain-use efficiency based on total net primary productivity.

In terms of RUE based on aboveground net primary productivity (RUE_ANPP_), no significant interaction between year and site was observed (Table 3). RUE_ANPP_ varied from 0.28 to 0.71 g m^−2 ^mm^−1^ with the lowest values for site HG for all years (Table 4). For each site RUE_ANPP_ was highest in the dry year 2005 and lowest in 2006. RUE based on total net primary productivity (RUE_NPP_) varied between 0.60 and 1.86 g m^−2 ^mm^−1^ with a significant interaction between year and site (Table 3, 4). For each site, RUE_NPP_ was highest in 2005 and lowest in 2004, but RUE_NPP_ of site UG79 was not significantly different between 2004 and 2006, while RUE_NPP_ in 2006 was significantly higher than in 2004 at both sites WG and HG. A strong correlation between RUE_ANPP_ and RUE_NPP_ existed at sites UG79 and WG (UG79: *R^2^* = 0.673, *P*<0.001; WG: *R^2^* = 0.742, *P*<0.001), while the positive correlation between RUE_ANPP_ and RUE_NPP_ was less pronounced at site HG (*R^2^* = 0.306, *P* = 0.029) (Figure 2). Regarding the year’s effect, both RUE_ANPP_ and RUE_NPP_ were highest in the dry year 2005, however we found contradictory results for the years 2004 and 2006. RUE_ANPP_ was higher in 2004 compared to 2006 but RUE_NPP_ showed the opposite results by being higher in 2006.

**Table 3:** F values and the probability (*P*> F) of two factored ANOVA results on RUE_ANPP_, RUE_NPP_, TE_ANPP_ and TE_NPP_.

Sources	Df	RUE_ANPP_	RUE_NPP_	TE_ANPP_	TE_NPP_
Y	2	25.89**	38.95**	30.51**	25.45**
S	2	8.50*	16.87*	7.02*	2.00 ns
Y * S	4	1.99 ns	2.74*	2.40 ns	6.37**

RUE_ANPP_ = rain use efficiency based on aboveground net primary productivity; RUE_NPP_ = rain use efficiency based on total net primary productivity; TE_ANPP_ = transpiration efficiency based on aboveground net primary productivity; TE_NPP_ = transpiration efficiency based on total net primary productivity; Y = year, S = site. Df = degrees of freedom.

* *P*<0.05,

** *P*<0.01 and ns: no significant difference.

**Table 4:** Differences between grassland sites and years in rain-use efficiency (RUE, g m^−2 ^mm^−1^) based on aboveground-(ANPP) and net primary productivity (NPP) in 2004, 2005 and 2006.

Year	UG79	WG	HG	Year ME
**RUE_ANPP_ (g m^−2^** **mm^−1^)**				
**2004**	0.54±0.02	0.39±0.01	0.37±0.02	**0.44±0.02 b**
**2005**	0.71±0.07	0.63±0.02	0.49±0.02	**0.61±0.03 a**
**2006**	0.39±0.02	0.35±0.01	0.28±0.10	**0.34±0.01 c**
**Site ME**	**0.54±0.04 x**	**0.46±0.03 x**	**0.38±0.02 y**	
**RUE_NPP_ (g m^−2^ mm^−1^)**				
**2004**	1.01±0.06 bx	0.69±0.04 cy	0.60±0.07 by	**0.79±0.06**
**2005**	1.86±0.09 ax	1.53±0.03 ay	1.17±0.04 az	**1.51±0.05**
**2006**	1.14±0.06 bx	0.98±0.02 by	0.79±0.04 bz	**0.97±0.04**
**Site ME**	**1.38±0.09**	**1.10±0.08**	**0.88±0.06**	

Significant differences in RUE_ANPP_ and RUE_NPP_ among years within a site are indicated by letters a-c and significant differences among sites within a year by letters x–z, respectively. Error: ± SE. Symbols for different sites as in Table 1.

Transpiration efficiency (TE_ANPP_) varied from 0.83 to 1.90 g m^−2 ^mm^−1^ and showed no significant interaction between year and site (Table 3, 5). TE_ANPP_ of site HG was significantly higher than that of sites UG79 and WG, while there was no significant difference between sites UG79 and WG. TE_ANPP_ in 2005 was significantly higher than that in 2004 and 2006. Although TE_ANPP_ in 2006 tended to be higher than it was in 2004, there was no significant difference between the two years.

**Table 5:** Transpiration efficiency (TE, g m^−2 ^mm^−1^) based on aboveground-(ANPP) and net primary productivity (NPP) of the three grassland sites in 2004, 2005 and 2006.

Year	UG79	WG	HG	Year ME
**TE_ANPP_ (g m^−2^** **mm^−1^)**				
**2004**	1.00±0.04	0.83±0.01	0.97±0.06	**0.94±0.03 b**
**2005**	1.56±0.16	1.53±0.05	1.90±0.09	**1.66±0.07 a**
**2006**	0.91±0.04	0.95±0.03	1.33±0.05	**1.06±0.05 b**
**Site ME**	**1.16±0.09 y**	**1.12±0.07 y**	**1.44±0.09 x**	
**TE_NPP_ (g m^−2^ mm^−1^)**				
**2004**	2.01±0.11 cx	1.47±0.09 cy	1.55±0.18 cy	**1.67±0.13**
**2005**	4.10±0.20 ay	3.72±0.07 ay	4.56±0.16 ax	**4.13±0.14**
**2006**	2.64±0.14 by	2.64±0.04 by	3.77±0.17 bx	**3.02±0.12**
**Site ME**	**2.99±0.21**	**2.73±0.20**	**3.49±0.28**	

Significant differences in TE_ANPP_ and TE_NPP_ among years within a site are indicated by letters a-c and significant differences among sites within a year by letters x–z, respectively. Error: ± SE. Symbols for different sites as in Table 1.

TE_NPP_ varied from 1.47 to 4.56 g m^−2 ^mm^−1^ and showed significant interaction between year and site (Table 3, 5). TE_NPP_ was not significantly different among three sites in 2004, while TE_NPP_ of site HG was significantly higher than that of sites UG79 and WG. The dry year 2005 significantly increased TE_NPP_ at all sites compared to the years 2004 and 2006.

**Figure 3:**
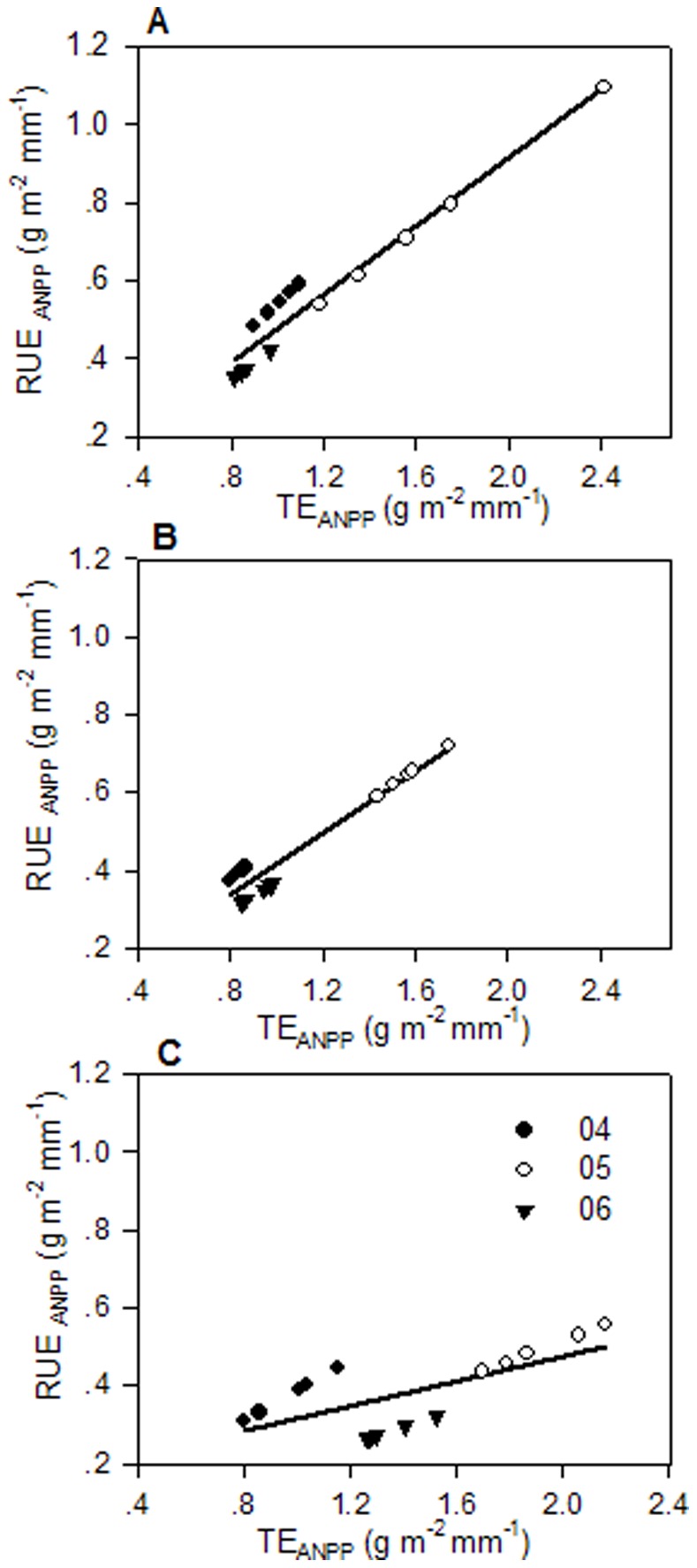
Relationship between RUE_ANPP_ (g m^−2 ^mm^−1^) and TE_ANPP_ (g m^−2 ^mm^−1^). Site UG79 (A), site WG (B), and site HG (C). The data for each site were from 2004, 2005 and 2006. The regression equations for UG79: y = 0.040+0.438 x, r^2^ = 0.9446, n = 15, P<0.001; WG: y = 0.024+0.393 x, r^2^ = 0.9387, n = 15, P<0.001; HG: y = 0.160+0.158 x, r^2^ = 0.4750, n = 15, P = 0.004. TE_ANPP_ = Transpiration efficiency based on aboveground net primary productivity. Same symbols as in Figure 2.

**Figure 4:**
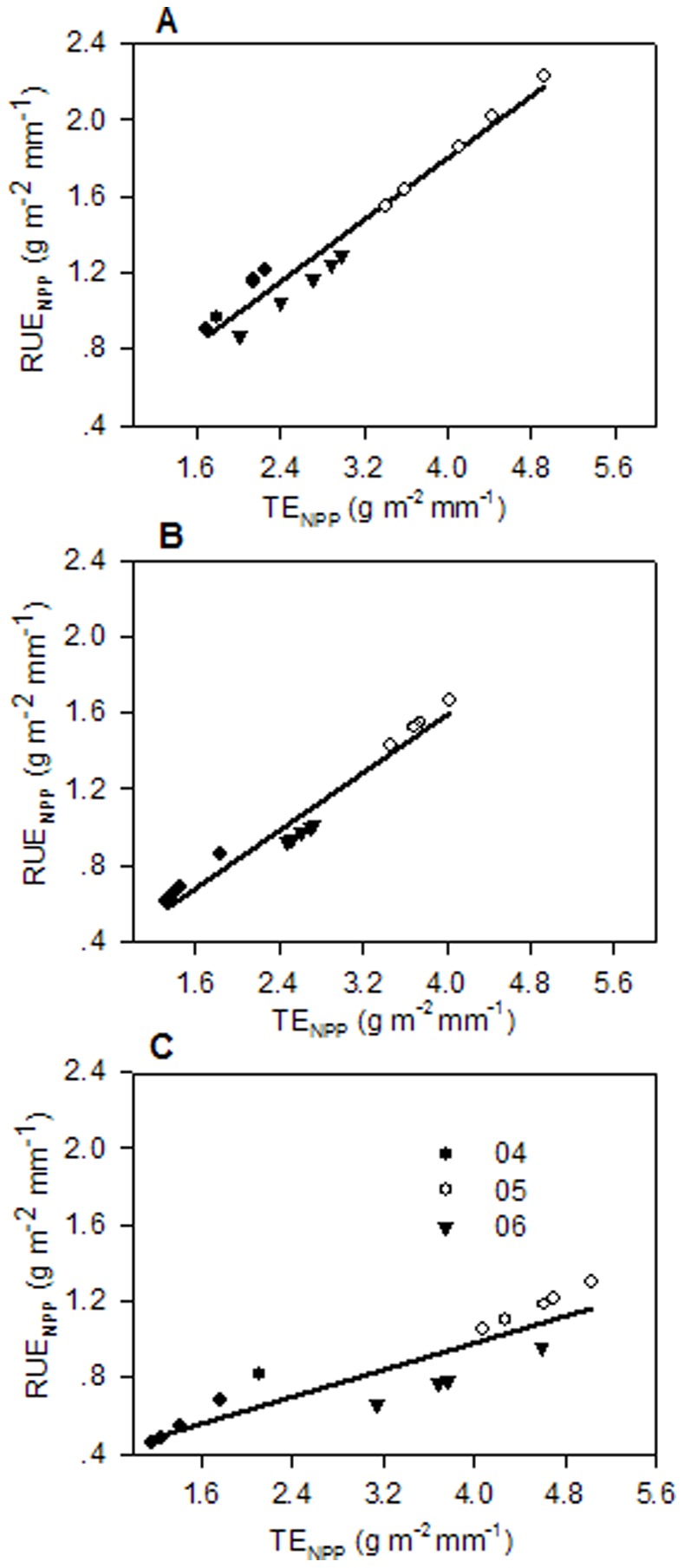
Relationship between RUE_NPP_ (g m^−2 ^mm^−1^) and TE_NPP_ (g m^−2 ^mm^−1^). Site UG79 (A), site WG (B), and site HG (C). The data for each site were from 2004, 2005 and 2006. The regression equations for UG79: y = 0.177+0.406 x, r^2^ = 0.9542, n = 15, P<0.001; WG: y = 0.084+0.370 x, r^2^ = 0.9626, n = 15, P<0.001; HG: y = 0.2734+0.176 x, r^2^ = 0.8055, n = 15, P<0.001. RUE_NPP_ = rain-use efficiency based on total net primary productivity; TE_NPP_ = transpiration efficiency based on total net primary productivity. Same symbols as in Figure 2.

**Figure 5:**
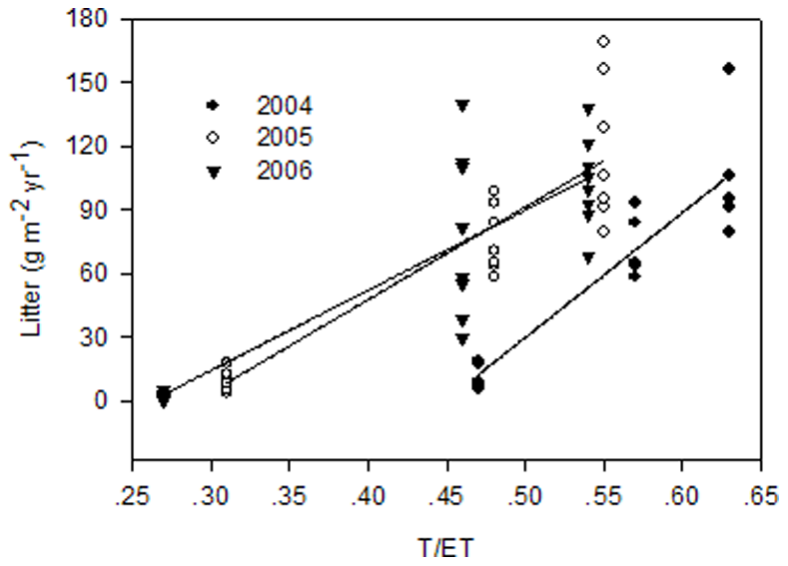
Relationship between litter amount and the ratio of transpiration to evapotranspiration. The regression equations for 2004: y = −263+587 x, r^2^ = 0.82 n = 15, P<0.001; for 2005: y = −126+435 x, r^2^ = 0.80, n = 21, P<0.001; for 2006: y = −98+377 x, r^2^ = 0.75, n = 24, P<0.001.

A strong correlation between RUE_ANPP_ and TE_ANPP_ existed at sites UG79 and WG (UG79: *R^2^* = 0.9446, *P*<0.0001; WG: *R^2^* = 0.9387, *P*<0.0001), while the correlation between RUE_ANPP_ and TE_ANPP_ at site HG (*R^2^* = 0.4750, *P* = 0.004) was not as strong and RUE_ANPP_ can only explain 47.5% variation of TE_ANPP_ (Figure 3). However, the correlation between RUE_NPP_ and TE_NPP_ was significantly improved when the calculation was based on total net primary productivity at site HG (*R^2^* = 0.8055, *P*<0.0001), and RUE_NPP_ explained the 80.55% variation of TE_NPP_ (Figure 4). At sites UG79 and WG transpiration efficiency was only slightly improved (UG79: *R^2^* = 0.9542, *P*<0.0001; WG: *R^2^* = 0.9626, *P*<0.0001) when the whole plant production was included (Figure 4). We found a strong positive correlation between litter amount and the ratio of plant transpiration to evapotranspiration (T/ET) in all three years (2004: *R^2^* = 0.82, *P*<0.0001; 2005: *R^2^* = 0.80, *P*<0.0001; 2006: *R^2^* = 0.75, *P*<0.0001) (Figure 5).

### Land use and Drought Effects on Root Distribution

**Figure 6:**
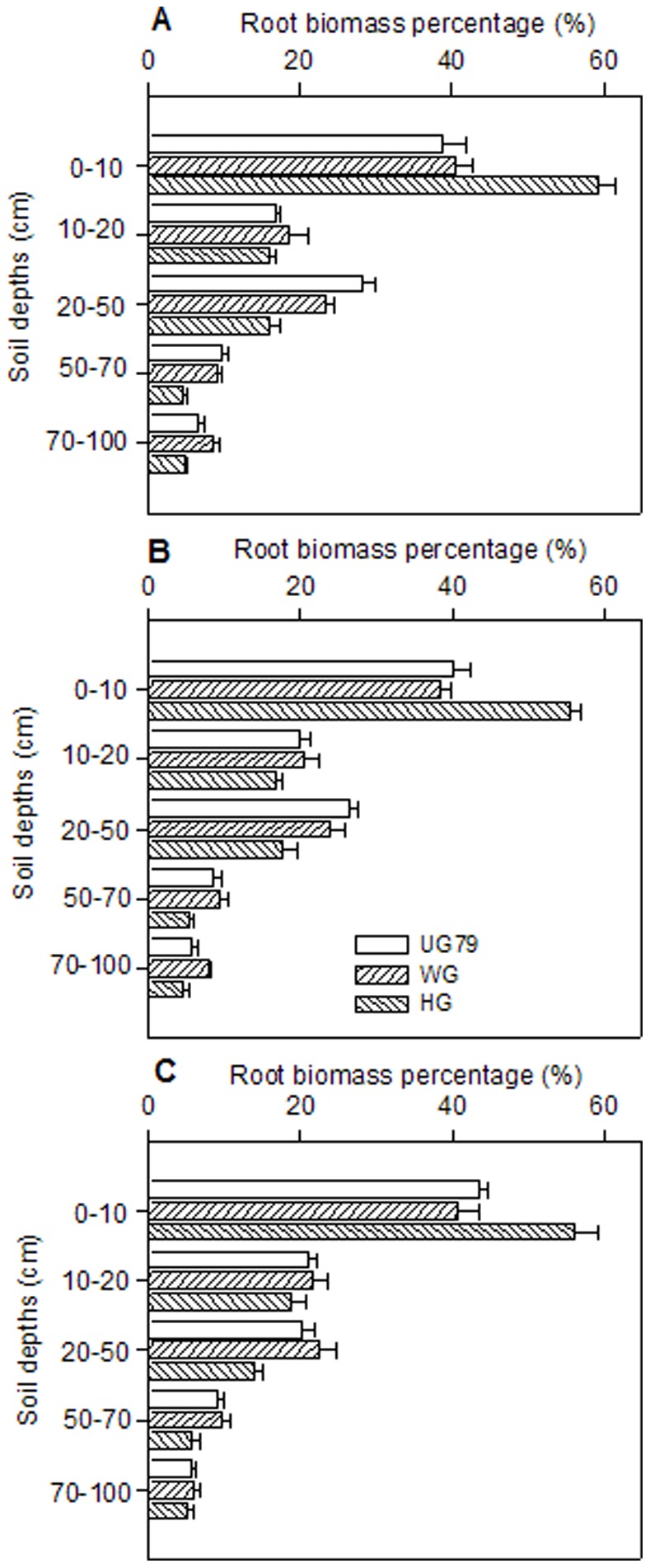
Relative distribution of root dry mass (%) in soil depths 0–100 cm. 2004 (A), 2005 (B), and 2006 (C). The data of each year were from three differently managed sites. Error bars represent mean ± SE. Same symbols as in Figure 2.

Overall, the distribution pattern of roots along soil profiles showed a similar trend for all sites (Figure 6), that is root biomass decreased with increasing soil depths and most of the roots were allocated in the top soil layer (0–20 cm). However, approximately 60% of the roots were allocated in the first 10 cm at site HG, whereas only approximately 40% of the roots were allocated in the same depth at sites UG79 and WG. Consequently, the relative root allocation to deeper soil layers was significantly lower at site HG than at sites UG79 and WG.

### Species δ^13^C Values and their Responses to Land use Intensity

**Figure 7:**
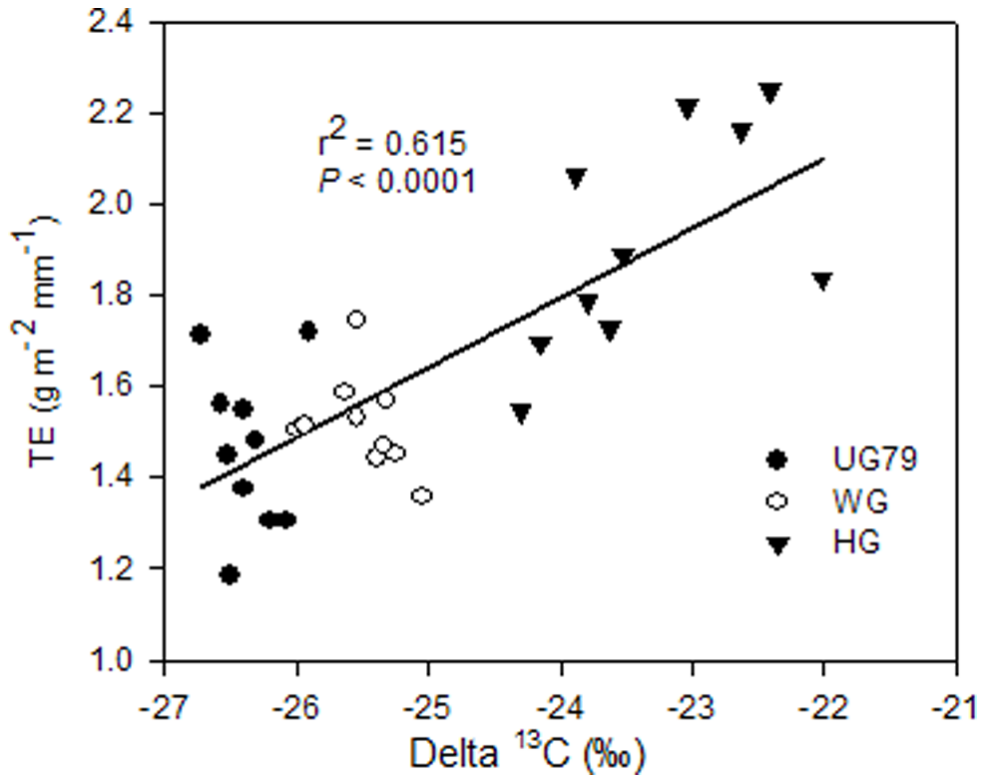
Correlation between transpiration efficiency and carbon stable isotope discrimination. The data were from the vegetation growth period of 2005 at three differently managed sites. Same symbols as in Figure 2.

As indicated by δ^13^C values, species exhibited different signatures in response to land use modes (Table 6). As for *Leymus chinensis*, *Stipa grandis* and *Artemisia frigida*, sites showed no significant effects on δ^13^C values, while significantly less negative δ^13^C values of *Cleistogenes squarrosa* and *Potentilla acaulis* were observed at site HG. Using bulk samples of shoot biomass pooled over species and sites, TE and δ^13^C showed a strong positive correlation (*R^2^* = 0.615, *P*<0.0001) (Figure 7).

**Table 6:** Carbon isotope discrimination (δ^13^C ‰) (mean ± SE) of dominant species at three differently managed grassland sites in July 2005.

Species	UG79	WG	HG	Chi-squared	Df	P-value
Lc	−26.17±0.08	−26.44±0.16	−26.60±0.15	3.78	2	0.1511
Sg	−26.34±0.09	−26.32±0.08	−26.22±0.09	0.78	2	0.6771
Cs	−14.48±0.03 b	−14.47±0.09 b	−13.74±0.08 a	9.43	2	0.0090
Pa	−29.48±0.19 c	−28.03±0.05 b	−26.96±0.07 a	12.5	2	0.0019
Af	−29.43±0.18	–	−29.13±0.12	1.32	1	0.2506

– not found.

Lc = *Leymus chinensis*; Sg = *Stipa grandis*; Cs = *Cleistogenes squarrosa*; Pa = *Potentilla acauli*; Af = *Artemisia frigida*. Kruskal-Wallis test was performed to show the significant difference among sites. Different small letters (a–c) indicate significant differences among sites within one species. Symbols for different sites as in Table 1.

## Discussion

### Effects of Land use and Drought on Rainfall use Efficiency

Land use effects on water use efficiency (WUE) have not been researched to a great extent but results, which mainly report on rain use efficiency (RUE), indicated decreasing RUE at degraded sites as compared to less degraded or even undisturbed sites [15]–[16]. However, a decrease of RUE in grazed grasslands is not generally a unique response, since positive effects of grazing on RUE were found in more mesic environments [17], [30]. Such an increase of RUE under grazing was mainly explained by higher photosynthetic activity of new leaves after defoliation, thus leading to increasing plant growth rates and ultimately over-compensatory growth.

Our results showed that grazing in this ecosystem did not stimulate plant growth and therefore reduced RUE at the more intensively used sites WG and HG compared to the long term protected site UG79. Increases of unproductive water loss by runoff and high bare soil evaporation on degraded or intensively used grassland sites have been viewed as the most important reasons for low RUE [7], [9], [15]. Le Houérou [7] reviewed the fact that the magnitude of evaporation from soil surface in arid and semi-arid rangelands varies from 20 to 70% of the infiltrated rain. Our results fell in the reported range with the highest values of up to 63.6% at site HG and the lowest values of 37.8% at site UG79 (Table 2). The strong correlation between litter biomass and the proportion of water lost by evaporation suggests litter as key factor in terms of water loss partitioning between plant transpiration and soil evaporation under different land use modes in this ecosystem (Figure 5). The contribution of surface runoff to unproductive water losses was relatively low at our sites compared to previously published results reporting runoff losses of 10% of total annual rainfall for semi-arid grassland systems [15]. Besides topography, the variable total rainfall amounts and intensities will strongly affect this water pathway, which might gain importance for the Inner Mongolian grasslands in wetter years but certainly should be carefully considered at sloping sites.

The widely accepted view is that RUE increases with rainfall up to a 450–500 mm annual mean precipitation. Further increases in annual rainfall amounts for grasslands usually reduce RUE due to increasing unproductive water losses, e.g. via run-off and drainage [6], [9]. However, prevoius studies also showed that a dry year following a wet year usually has high RUE for a given site [2], [7]. Consistent with these findings, our results showed a higher RUE in the dry year 2005 than in 2004 at all sites. This was because ANPP decreased by 21–36% in 2005 compared to 2004, while rainfall amount was 52% less, which consequentially translates into higher RUE.

Several possible mechanisms could explain the increased RUE for water limited years. It is not likely that soil water storage and hence water availabilty footing on the previous year’s precipitation could be used in the subsequent dry year since very little ground water contributes to current evapotranspiration. Bai et al. [9] reported that ANPP during wet years increases more than it declines in corresponding dry years. Possible biological mechanisms have been observed in Inner Mongolian steppe plant species that accumulate photosynthate in storage organs during sufficient wet years, providing resources which result in relatively more growth in the subsequent dry year (unpublished data). However, the year 2006 did not show high RUE values but rather the lowest, indicating a depletion of carbohydrate reserves within the year 2005 under unfavourable growing conditions and carry-over affecting the growth in 2006. Furthermore, in 2006 considerable rain was received in September (Figure 1) out of the main growing season when most of plants were already dormant. These examples illustrate that rainfall distribution and carryover effects of drought events between years and plant phenological cycles need to be considered when analyzing productivity and water use efficiency of perennial semi-arid grasslands [4], [31]–[33]. Unsynchronized rainfall events in relation to plants’ water demand may lead to an increase of unproductive water loss, which was illustrated by the low RUE in 2006.

### The Relationship between RUE_ANPP_ and RUE_NPP_

Analysis of semiarid grassland ecosystem’s RUE as an indicator for ecosystem performance are almost exclusively based on aboveground net primary productivity due to the almost complete lack of root information required for the calculation of total plant net primary productivity [7], [9], [15], [34]. However, information on NPP and the whole plant water use efficiency is relevant in terms of quantifying the seasonal total net C and water flux [35]. Our results only partially support that RUE_ANPP_ can be used as a proxy for RUE_NPP_. At sites UG79 and WG, representing areas of maximum ecosystem conservation and moderate land-use intensity respectively, biomass partitioning between above- and belowground plant parts was relatively consistent over the three years with approximately 50–60% biomass located belowground [10] and thus RUE_ANPP_ was qualitatively reflecting RUE_NPP_ at these two sites (Figure 2). However, RUE_ANPP_ at site HG did not correlate very well with RUE_NPP_ due to inconsistent biomass partitioning among shoot and root systems within the different years (Figure S1). It was likely that heavy grazing induced complex and inconsistent feedback between soil nutrients, soil water and other soil physical factors with species composition [15], [36]–[37]. We suggest that at intensively used grassland sites the whole plant production should be taken into account for elucidating RUE_NPP_. Consequently, meta-analysis of RUE data based on ANPP should be carefully considered in case heavily grazed sites are involved. These findings futher underline the relevance of in-depth analysis of BNPP dynamics in grassland systems. C-flux estimates, which rely on aboveground biomass information, may tend to underestimate the carbon uptake capacity of grazed sites in relation to water use.

### Transpiration Efficiency (TE)

No systematic studies analyzed the land use effects on plant transpiration use efficiency (TE) using field-based ecological data. This lack of data is attributed to difficulties in partitioning water losses via evapotranspiration (ET) into E and T within water balance equations. However, analyzing TE of ecosystems will provide essential information about plant performance under variable environmental stress and thus will allow the discussion of adaptive traits and the resilience potential of plant communities.

Both TE and RUE efficiency increased under water limited conditions at all three sites, and both showed the carryover effect for 2006 with relatively low indices compared to 2005. Comparing the values of RUE and TE for different sites, RUE_ANPP_ correlated well with TE_ANPP_ at sites UG79 and WG but not at site HG (Figure 3). However, when the whole plant productivity was considered, the linear correlation for site HG improved considerably (Figure 4), indicating that water use efficiency at site HG tends to be underestimated when only the aboveground part is considered. Notably, slopes of the regression decreased with increasing land use intensity indicating that transpiration efficiency increased relatively compared to RUE under high land use intensity. In this context, it was surprising to see that the plant community at site HG showed the highest TE in the dry years 2005 and 2006 for both ANPP and NPP based analysis while having the lowest RUE at the same time (Table 4, 5).

There are several plant-related aspects which might help to explain this offset phenomenon between RUE and TE under intensive land use. Efficient water use is viewed as one of the key factors of adaptability of natural vegetation to general water limitations. Firstly, the abundance of C4 species was high at site HG, with a relative biomass ranging from 15% to 24%, while C4 species were rather rare at site UG79 [4]. *Cleistogenes squarrasa* was the most common C4 species at site HG since it has strong tillering ability, high photosynthetic capacity and an acknowledged high WUE [38]–[40]. This species may efficiently utilize small rainfall events due to its fine, dense and shallow rooting system (Field observation). Relevance of the ability to use small rainfall events as a strategy has been proved in shortgrass steppe by *Bouteloua gracilis*, a small C4 bunchgrass [41]. Secondly, significant increases of opportunistic species, like *Chenopodium spp*., can also contribute to high TE as these species can grow very rapidly when nutrients and water are readily available [13]. Thirdly, as indicated by values, the “grazing increaser” species *C. squarrasa* and *Potentilla acaulis* enhanced WUE with increasing grazing intensity, while the WUE of the “grazing decreaser” species *Leymus chinensis* and *Stipa grandis* has no significant difference among sites (Table 6). Fourthly, smaller rainfall events with low infiltration depth often ocurred in this semi-aridecosystem [11], providing water available to plants only in the very topsoil. Plants developing a dense rooting system in the topsoil (Figure 6) are thus outcompeting species with deeper rooting systems and can take up water which would be lost by evaporation and therefore contribute to higher WUE. Furthermore, the possible reasons for these species differences in WUE remain speculative but must be related to differences in root system size and allocation strategy along the soil profile. This argument is based on the concept that the shallow root systems are generally favoured over deep root systems mainly because energy costs for construction, maintenance and resource uptake are lower for shallow roots [42]. This is exactly the case when plants grow under combined stresses of frequent water shortage and heavy grazing. These adaptive mechanisms of efficient water use at heavy grazing sites could be a key trait of high resilience to multiple stresses in semi-arid ecosystems under intensive land use. To analyze WUE of different land use types in this steppe ecosystem, the relevance of transpiration efficiency and total plant productivity appears obvious to avoid a systematic underestimation of intensively used grassland sites [10].

## Conclusions

The analyzed grassland system shows a high response to grazing and rainfall variability in terms of primary productivity and water use efficiency. All analyzed sites representing different land use modes increased RUE under water limiting conditions. Although our measurements indicate decreased plant transpiration and consequently decreasing RUE under heavy grazing, productivity was relatively compensated with higher transpiration efficiency. This was even enhanced under water limited conditions and more evident when including belowground production. From our data we suggest considering the whole plant perspective by adding root systems into WUE index when intensive land use sites are concerned. RUE and aboveground net primary productivity could only partially function as a proxy for transpiration efficiency and whole plant net primary production respectively. Transpiration efficiency rather than RUE can be as a functional indicator to identify site specific plant community functional traits related to water use efficiency. Both the consideration of the whole plant perspective and “real” water use efficiency would partially revise our picture of system performance under different rainfall regimes and land use practices and therefore certainly affect the discussion on the C-sequestration and resilience potential of ecosystems.

## Supporting Information

Figure S1 Productivity (g m^−2^ yr^−1^) of three differently managed grassland sites. Above net primary productivity (A), belowground net primary productivity (B), and total net primary productivity (C). Error bars represent mean ± SE.
(TIF)
