# Supplementary figures and images for: Community Level Offset of Rain Use- and Transpiration Efficiency for a Heavily Grazed Ecosystem in Inner Mongolia Grassland
Source: PLoS One. 2013 Sep 18;8(9):e74841. doi: 10.1371/journal.pone.0074841 (PMC3776764; doi:10.1371/journal.pone.0074841)

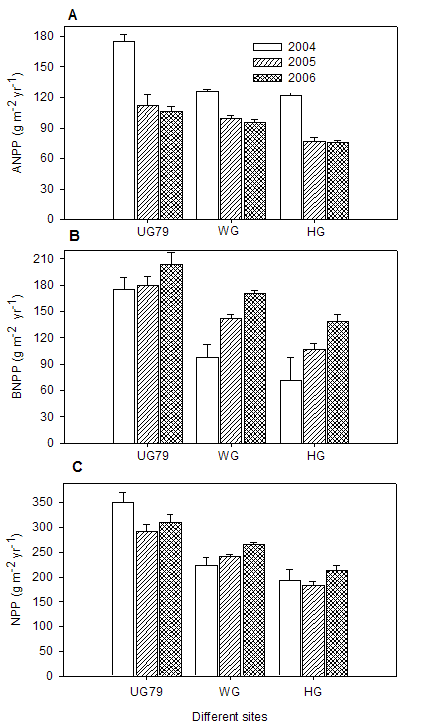

Supplement: Figure S1 — Productivity (g m−2 yr−1) of three differently managed grassland sites. Above net primary productivity (A), belowground net primary productivity (B), and total net primary productivity (C). Error bars represent mean ± SE. (TIF) [file pone.0074841.s001.tif]
